# Adverse incidents and therapy options for opioid use disorders in forensic psychiatry

**DOI:** 10.1192/j.eurpsy.2023.1131

**Published:** 2023-07-19

**Authors:** A. Voulgaris

**Affiliations:** Institute for Sexual Medicine and Forensic Psychiatry, Universitätsklinikum Hamburg Eppendorf, Hamburg, Germany

## Abstract

**Introduction:**

Patients admitted into Forensic Clinics for Dependency Diseases (FCDD) in Germany are diagnosed with at least one substance use disorder. Opioid use disorders is common in this clinical population. Surprisingly, data on the availability and practice of opioid substitution treatment (OST) options in German FCDD according to Sect. 64 of the German Criminal Code (StGB) is scarce. Additionally, important data on the prevalence of adverse incidents such as violent behavior, relapse or escape from the clinic are missing for this highly specific treatment setting.

**Objectives:**

Our aim ewas to describe the clinical practice regarding opioid substitution therapy in forensic psychiatry in Germany and to identify the prevalence of relevant adverse incidents during the therapy process.

**Methods:**

We conducted a observational study including all FCDD units in Germany via a questionnaire. We assessed the clinical practice and total number of patients that received an OST, clinical reasons for beginning and ending the OST, number of treatments terminated without success, number of successful treatments and relevant adverse incidents such as violent behavior, relapse, escape and reoffending. The data was analyzed descriptively.

**Results:**

15 of the existing 46 FCDD participated in our study (33%). In total, 2483 patients were treated in the participating FCDD, 18% of the patients were relocated into prison due to treatment termination and 15% were discharged successfully. 275 adverse incidents were reported: violence against a patient (4%), violence against staff (1,6%), escape (4,7%) and reoffending (0,5%). Merely in seven FCDD treating 1153 patients, an OST was availabe. Available options included buprenorphine/naloxone, buprenorphine, methadone and levomethadone. Regarding adverse incidents and successful discharge, no differences were detected in the clinics with or without an OST. In the clinics that offered an OST, we found a significantly higher rate for treatment termination without success (p<0.007) in comparison to clinics without this program. 99 patients received an OAT and this treatment was ended due to illegal drug abuse (57%), refusal to give an urine drug sample (71%) and in cases were the OAT was given away to other patients (85%).

**Conclusions:**

Surprisingly, opioid substitution therapy is only accessible in a part of the FCDD in Germany. Reasons for this are unclear. Critical incidents such as violent behavior against staff/patients and escape are not uncommon in this forensic psychiatric treatment setting. Further studies are needed to enhance the understanding of the limited OST practice and the risks for patients and staff in this specific forensic treatment setting.

**Disclosure of Interest:**

None Declared

